# Supplementary material for: A Whole-Genome Sequencing Study Implicates GRAMD1B in Multiple Sclerosis Susceptibility
Source: Genes (Basel). 2022 Dec 16;13(12):2392. doi: 10.3390/genes13122392 (PMC9777893; doi:10.3390/genes13122392)
Supplement: Supplementary file 1 [file genes-13-02392-s001.zip › genes-2067659-supplementary.pdf]

A Whole-Genome Sequencing Study Implicates GRAMD1B in Multiple Sclerosis Susceptibility

Supplementary Tables and Figures

Table S1. Sequences of primers used for target sequencing. The exon numbers and expected amplicon sizes are shown.

Exon number	Chr	Sequence primer forward	Sequence primer reverse	bp of amplicon
1	11	GCTCAGAGCTGGTATGAGGTG	CAGAAGAACTGCTGCCACTG	246
1	11	GAATCTGTCCTTTGCCTCCA	GGTGCCCTATCCCTCCTCTA	285
2	11	ACTGACTGCCTGCCTTTCC	GGCTCACCACCCCAATATC	349
3	11	ACCACATGGGAAGCATTCAG	CCAACAAGAAGGGAAACAGG	188
4	11	CTTGCCCTTCCTTCCTAACC	ACCCTGAGTGCTATGCCG	219
5	11	CAGTGGTTTGCCGAAAATG	GCAGCTTGCTCAGCCATAG	222
6	11	CCTGACTTACTAATGCCCCAC	ATGCGATTCAAGAAAGGGC	236
7	11	TTTTCTTAAAGGATGGAGGACC	TGGGAGAATACTATCAGTGGGG	268
8	11	CCTCTGTGCAGAGTCGGG	TCTTCCTGCAGGCACTCG	236
9	11	GGATGGATAGAAAGGTCTGAGATTC	GAAACGCTGGCCACAGG	297
10	11	TCCCTTTGGAGAATAGATCCAG	CCACATAGAGATCTCCTTTTAGCC	325
11	11	CTCTTGGAGGATGTCCCTGG	TGTAGAGAGGGAAGAAGTGAGG	284
12	11	AAACTTGGATTGGGGAGAGG	TGCCCTTAATCCTTCAAGACAG	256
12	11	AGGGGAAACTTGGATTGGGGA	TCCTGACATCTGCCCTTAATCCT	271
13	11	TCCAAGCTTCTTGCTCCTC	CTCCTCCTTCCCCGTCAG	282
14	11	AATTTCCCTTTTCCCAGCAG	CCGCAGTGCAGTTTACCATC	233
15	11	TGCAGAGAGTTGCATTGGAG	GTGTGTGCCTTGGTGTGG	341
16	11	ACCAGCAGTTGTTGGACTTG	CTTCAAGAGACGAGGGCTGG	231
17	11	TTTTCTTGTGCCCTGTCCTT	GGGGAGGAAAAGAGATGAGG	240
18	11	CAAGGAGACTGGGAGACAGG	GGGATGTCCTAGGCTCAGGT	247
19	11	GTCCTTTAGGGGTCTGGGAG	GGAGGGTGGTGATAGAGACG	297
20	11	AATCCCACTAAAAGCGCAGA	CCTGTTCCTGCCTTGTCAAT	158
20	11	GGAGCAGCGTTTTCTTATGG	TGGCTCAACATTTCCCTTCT	150
20	11	ATTGACAAGGCAGGAACAGG	TCAGCTGCTCCACCATAAGA	222

Table S2. Sequences of primers used for qRT-PCR. The target genes and sequences of primers used in qRT-PCR are shown.

		Sequence (5′ to 3′)	Reference
hGRAMD1B	Forward	GTGGACTGGAGGACTACTTC	Designed in-house
	Reverse	GTTAAGGATGACCAGCAGCA	
hGAPDH	Forward	CATGTTCGTCATGGGTGTGA	Designed in-house
	Reverse	AGTCTTCTGGGTGGCAGTGA	
rGramd1b	Forward	GTGGACTGGAGGACTACTTC	Designed in-house
	Reverse	GTTAAGGATGACCAGCAGCA	
rFrataxin	Forward	TCACCATTAAGCTGGGCG	Pelizzoni et al. (2012)
	Reverse	TTCTTCCCGGTCCAGTCATA	
rRpI18S	Forward	CAGAAGGACGTGAAGGATGG	Pelizzoni et al. (2012)
	Reverse	CAGTGGTCTTGGTGTGCTGA	

Table S3. Demographic and clinical features of MS patients and healthy controls used in the immunohistological characterization. Description of the characteristics of MS patients and healthy controls including the demographic, clinical features, disease duration at time of autopsy, concomitant disease-modifying drugs and the lesion/tissue type that was analysed. Based on the pathological analyses tissues were classified as follows: A=active lesion, I=inactive lesion, NAWM= non-affected white matter, NWM=normal white-matter. IFN 1β: Interferon β-1 a; GA: Glatiramer Acetate.

Patient	Age	Gender	Diagnosis	Tissue Analyzed	Tissue Classification	Disease Duration (yrs)	Disease-modifying drugs
**MS1**	50	Female	SPMS	Pons	I	13	Dalfampridine, Baclofen
**MS2**	54	Female	RRMS	Medulla	I, NAWM	17	Steroids, IFN 1β , GA
**MS3**	45	Female	SPMS	Pons	A, NAWM	7	Steroids, Phenytoin, Lorazepam
**MS4**	45	Female	PPMS	Pons, monocytes/myeloid monocytes/myeloid Medulla	A, NAWM	16	Baclofen
**C1**	41	Male	---	Pons	NWM	---	---
**C2**	69	Female	---	Occipital Cerebrum	NWM	---	---
**C3**	81	Male	---	Pons	NWM	---	---

Table S4. Variants emerged from Canadian MS families. Genomic coordinates are given from NCBI Build 37.1; RNA and protein positions are based on GenBank RefSeq NM_020716.2; dbSNP IDs are provided from build 149. Exome Aggregation Consortium (ExAC) frequencies were obtained from European non-Finnish samples, with an asterisk indicating low quality data. Damage prediction on protein function and evolutionary conservation were calculated using Combined Annotation Dependent Depletion (CADD v1.3) phred-scale and Genomic Evolution Rate Profiling (GERP). Pos: position; nt: nucleotidic.

pos	nt change	cDNA change	Amino Acid change	dbSNP	CADD	GERP	Genotypes	MS patients (MAF)	Controls (MAF)	ExAC (MAF)
11:123479372	G/A	c.1090G>A	p.V364I	rs200540342	23.1	5.52	GG/GA	9/2421 (0.19%)	3/1061 (0.14%)	70/7402 (0.46%)*
11:123484247	C/T	c.1679C>T	p.T560M	rs199604534	23.8	4.37	CC/CT	5/2426 (0.10%)	1/1062 (0.05%)	15/322163 (0.02%)

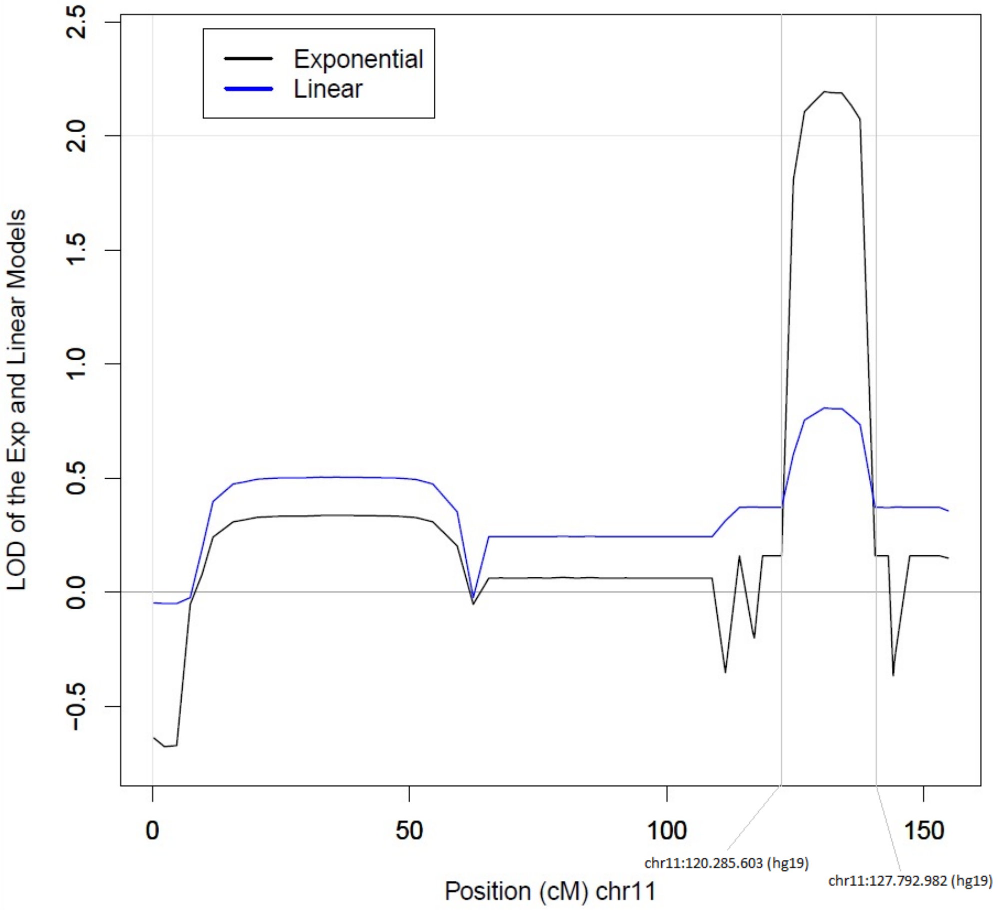

Figure S1. Linkage peak identified in chromosome 11q23. The peak identified in the linkage analysis is shown. The start and end positions of the peak are indicated on the x-axis. A linear and exponential model has been tested according to Kong and Cox.

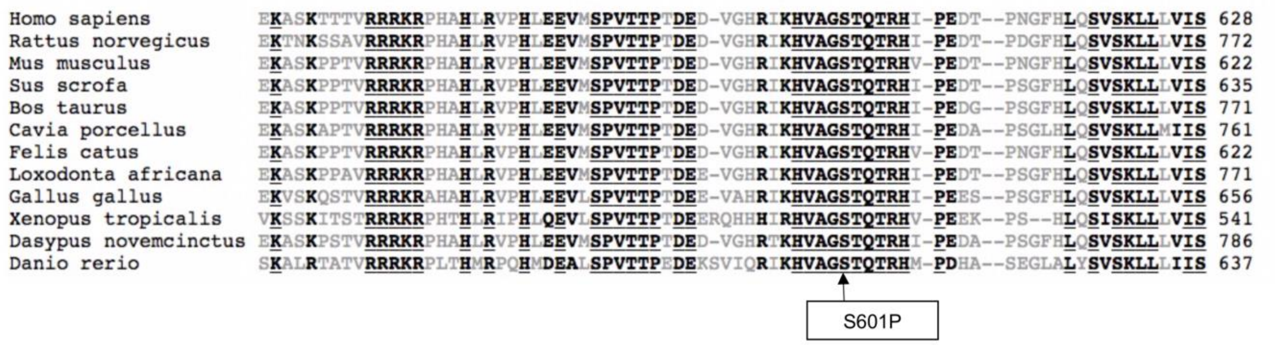

Figure S2. Conservation analysis of S601P. Multiple sequence alignment of GRAMD1B among vertebrates around p.S601P mutations (indicated with arrows) showed conserved amino acid change across species from zebrafish to humans.

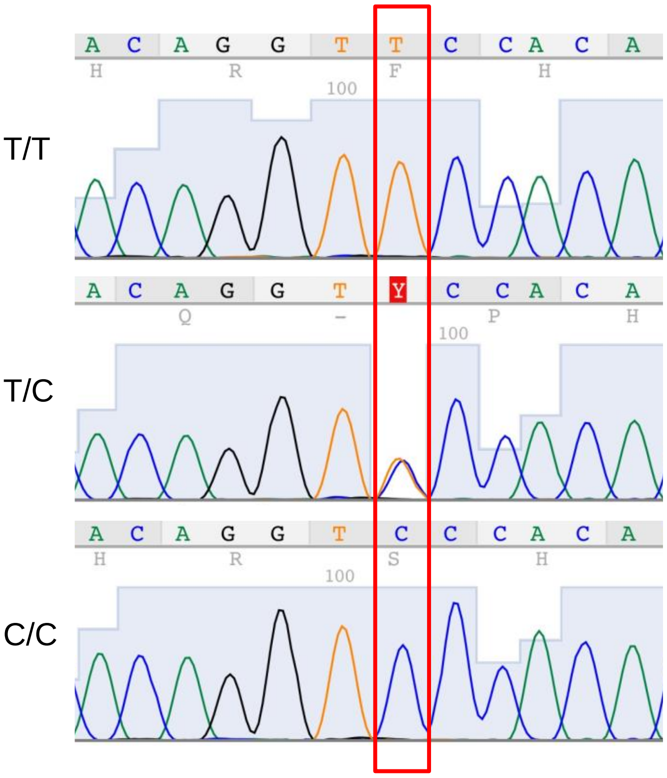

Figure S3. Sanger validation of the *c.1801T>C* (p.S601P) variant. Representative example of the electropherograms showing the presence or absence of the *c.1801T>C* (p.S601P) variant in a multiple sclerosis affected homozygous for wild type (subject: II-3), heterozygous (subject: III-5) and homozygous for the mutant allele (subject: II-6).

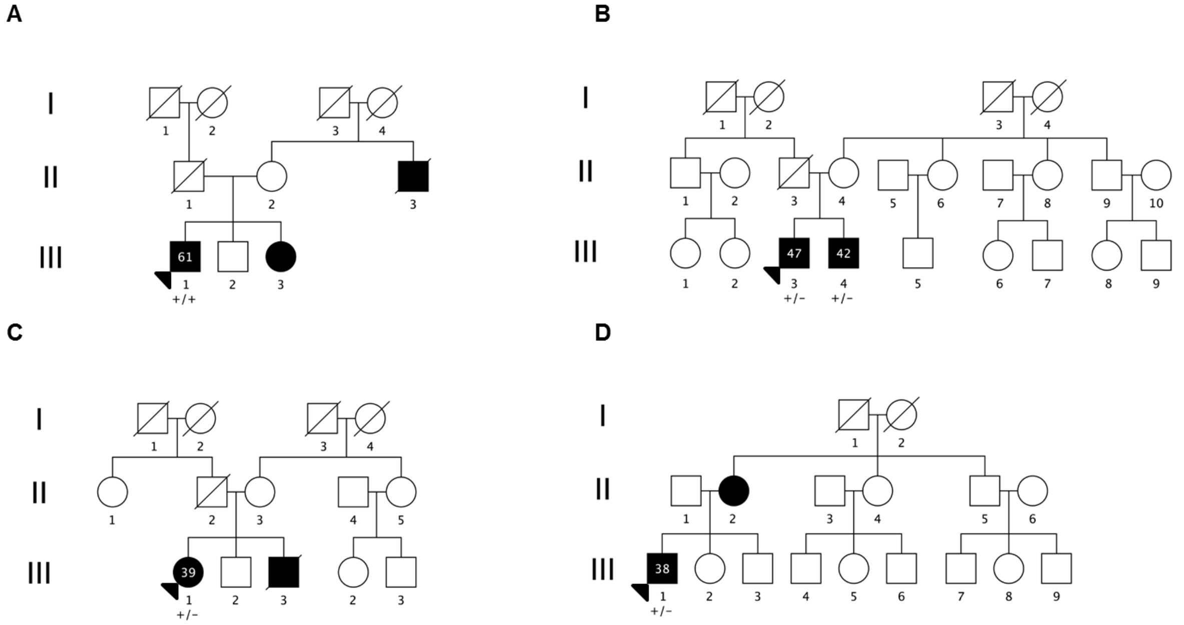

Figure S4. Pedigrees of the familial MS cases in which additional *GRAMD1B* rare variants have been identified. The pedigrees of MS multiplex families in which we found additional *GRAMD1B* rare variants are plotted. Specifically, in family A the proband carries the rs118067934 variant at homozygous state, in family B the two subjects affected by MS carry the rs140366389 variant at heterozygous state, in family C the proband is a carrier of the rs755488531 variant at heterozygous state, whereas in family D the proband is a carrier of the rs769527838 variant at heterozygous state. For an additional family, where the proband carries the rs118067934 variant, we did not have enough information to reconstruct the pedigree. The numbers reported inside circles and squares indicate the age of the subject at sampling. Squares represent men, circles represent women; slash, deceased subject. Black-filled symbols represent individuals diagnosed with MS. Small arrowhead, proband. +/+: homozygous individuals for mutated allele; -/-: homozygous individuals for wild-type allele; +/-: heterozygous individuals.

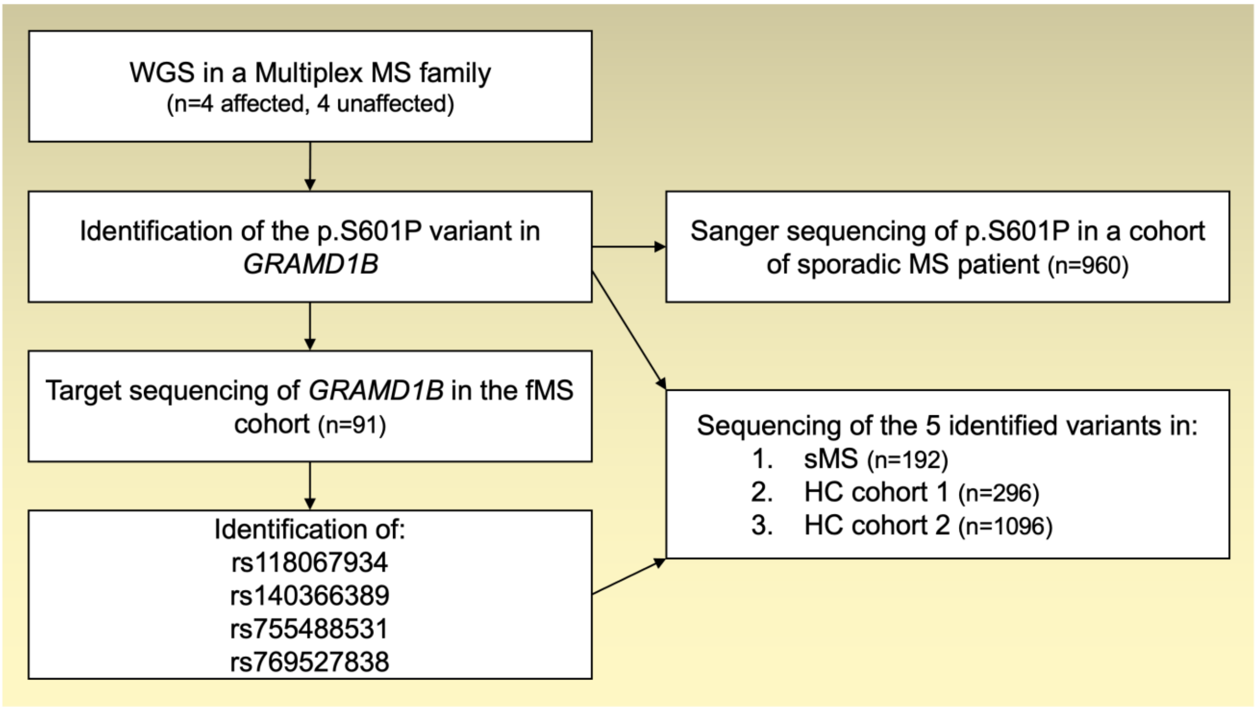

Figure S5. Summary flowchart of the different case-control data sets and the variants tested. The flowchart describes the different steps of the variant identification in *GRAMD1B* gene. Specifically, whole genome sequencing and subsequent variants filtering were performed in the described multiplex MS family leading to the dentification of the p.S601P variant in *GRAMD1B*. Then, the presence of the variant was tested in a cohort of 960 sporadic MS cases: none of the MS patients carried the p.S601P variant. A targeted sequencing approach was applied to re-sequence exons and exon-intron boundaries of *GRAMD1B* in 91 unrelated MS probands with at least one first or second degree relative affected by MS (fMS); leading to the selection of additional 4 rare variants in *GRAMD1B*. Finally, we then tested the presence of the identified 5 rare variants in an additional cohort of 192 sporadic MS cases (sMS) and 296 HC subjects (HC cohort 1), as well as in 1096 HC Italian subjects from the Italian Reference Genome v1 (HC cohort 2).

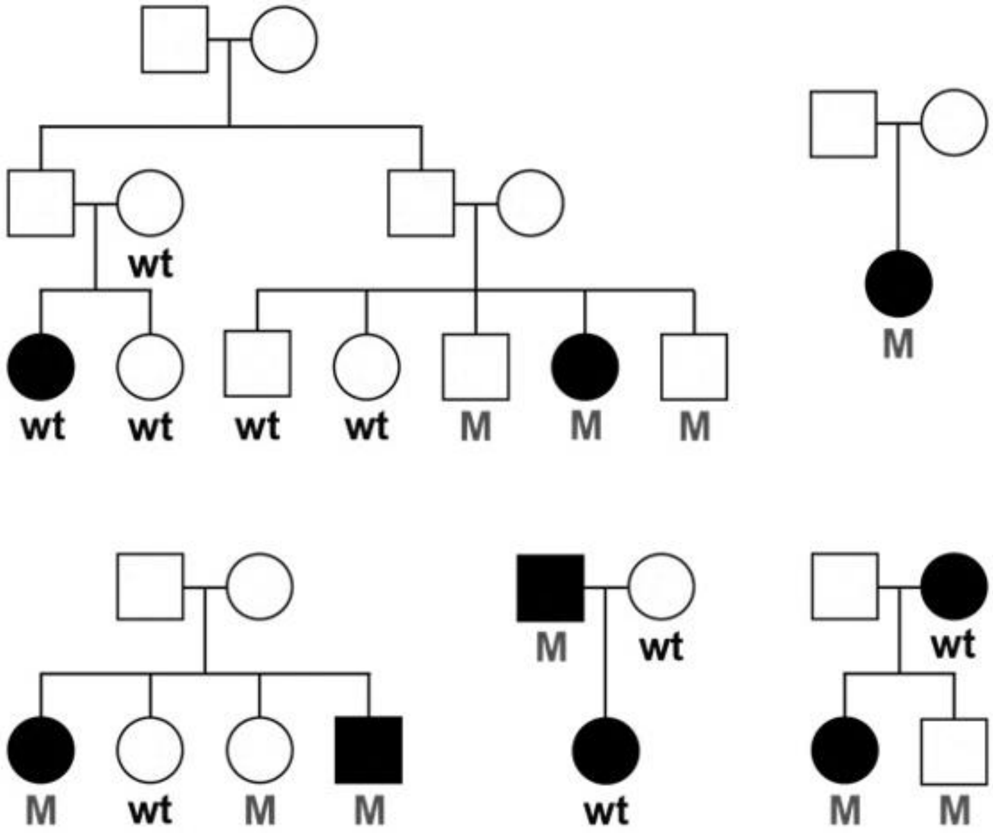

Figure S6. Pedigrees of the additional Canadian familial MS cases with included the information about the segregation of p.T560M. Cases are indicated in black. wt: wild-type, M: carrier of mutation.

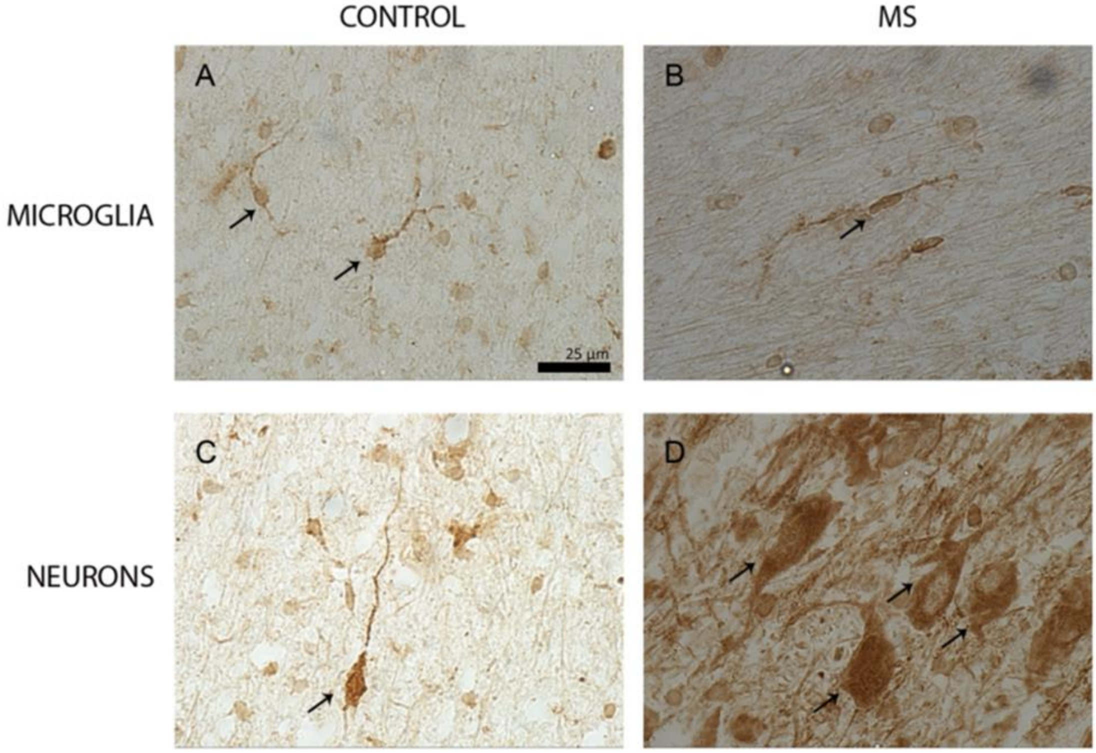

Figure S7. GRAMD1B expression in microglia and neurons in brains of MS patients and healthy controls. A-B: Representative DAB (3,3–diaminobenzidine) immunostaining with the GRAMD1B antibody in normal white matter (NWM) of a control subject (CTR) and in the normal appearing white matter (NAWM) of a MS patient respectively. Staining corresponds to microglia cells. C-D: Positive DAB staining in subcortical neurons of a control and in the pons of a MS patient respectively. Black arrows indicate the cells of interest.

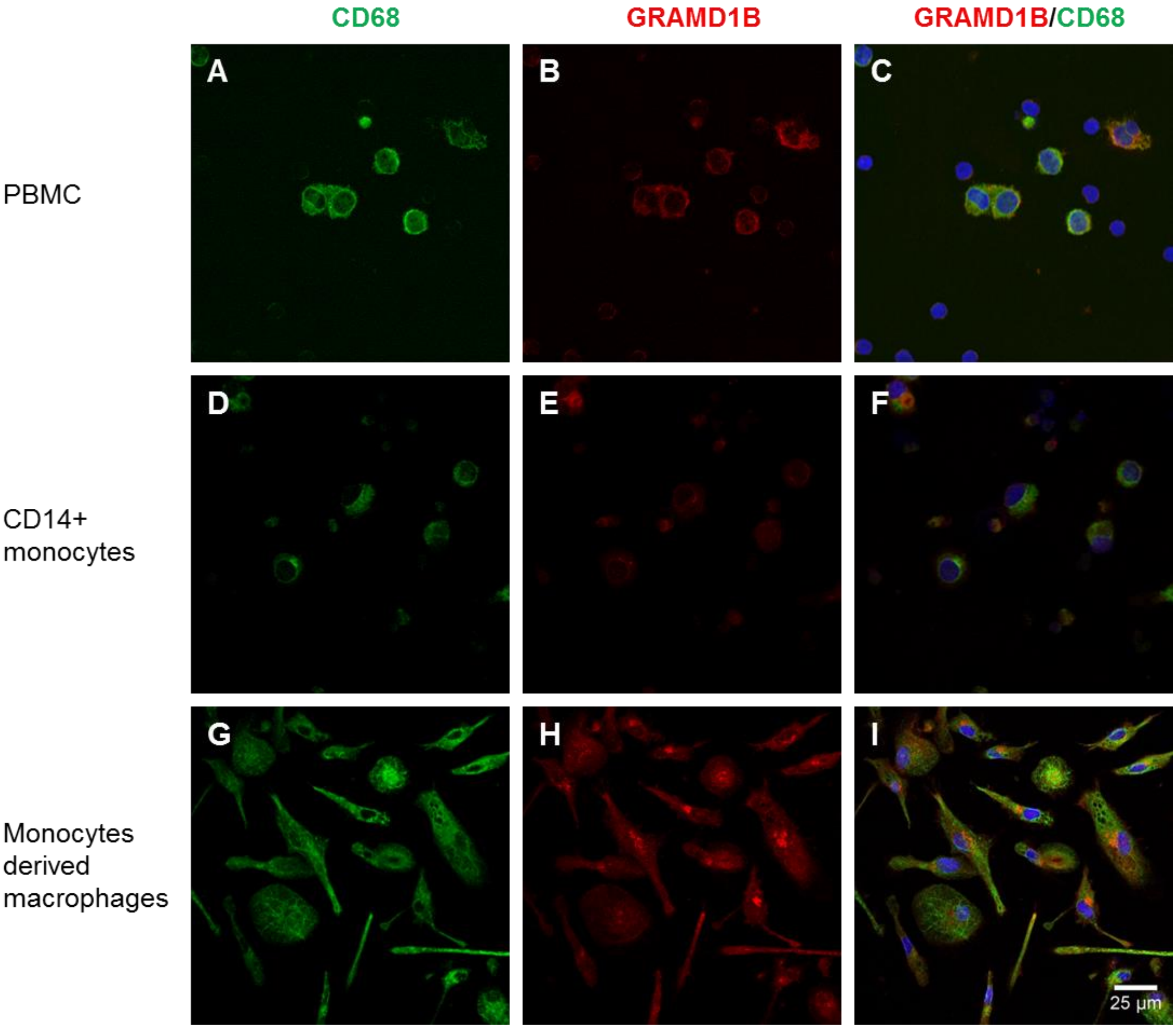

Figure S8. GRAMD1B expression in primary human immune cells isolated from peripheral blood of healthy controls. Immunocytochemistry of GRAM1B in PBMC (A-C), $CD14^+$ monocytes (D-F) and macrophages/myeloid ($CD68^+$) cells (G-I). GRAMD1B staining is shown in red, and the corresponding cell marker CD68 for monocytes and macrophages/myeloid cells is shown in green.
